# A Fresh Fruit and Vegetable Prescription Program for Prenatal Patients in Flint, Michigan: Baseline Food Security and Dietary Intake

**DOI:** 10.3390/nu16081234

**Published:** 2024-04-21

**Authors:** Amy Saxe-Custack, Jenny LaChance, Jean M. Kerver

**Affiliations:** 1 Charles Stewart Mott Department of Public Health, Michigan State University-Hurley Children’s Hospital Pediatric Public Health Initiative, Flint, MI 48502, USA; jlachan1@hurleymc.com; 2 Department of Epidemiology and Biostatistics, Michigan State University, East Lansing, MI 48823, USA; kerverje@msu.edu

**Keywords:** fruit and vegetable prescriptions, diet, pregnancy, food security, low-income, maternal, food access, fruits and vegetables

## Abstract

Although adequate nutritional status during pregnancy is necessary to support optimal fetal development, many low-income women have poor access to fresh, high-nutrient foods. To address these challenges, a pediatric fruit and vegetable (FV) prescription program was expanded to include pregnant women, providing one prescription for fresh FVs worth 15 US dollars during each prenatal office visit for redemption at farmers’/mobile markets. This analysis describes baseline sociodemographic characteristics, food security, and dietary intake among 253 pregnant women in Flint, Michigan in 2022–23. Dietary recall data were collected and analyzed using the Automated Self-Administered 24-h Tool developed by the US National Cancer Institute, with nutrition output reported in relation to adherence to US Dietary Guidelines. Most participants (mean ± SD age 26.51 ± 4.90 years) identified as African American (53%) and were receiving publicly funded health insurance (66%). Most (75%) reported no food insecurity, yet the majority failed to meet dietary recommendations for whole grains (99.3%), vegetables (93.1%), dairy (93.1%), and fruits (69.4%). Moreover, most did not meet micronutrient recommendations through food sources, including vitamin D (100%), iron (98.6%), folic acid (98.6%), vitamin A (82.6%), calcium (68.8%), and vitamin C (62.5%). Results raise deep concerns regarding diet and nutrition among pregnant women in this US city.

## 1. Introduction

Pregnancy is a vital period during which maternal nutrition and lifestyle choices are major influences on both women and child health [1,2]. Increasing evidence demonstrates that proper nutritional status during pregnancy is crucial to support optimal fetal growth and brain development as well as early prevention of adverse pregnancy outcomes, infant mortality, birth defects, and chronic diseases throughout the lifespan [3]. Unfortunately, there are numerous barriers to accessing high-nutrient foods in low-income communities, including limited full-service grocery stores, poor-quality fresh foods, lack of transportation, and financial constraints [4,5,6,7,8,9]. Moreover, many women struggle with competing priorities and lack of skills necessary to operationalize dietary recommendations during pregnancy [10]. 

In Flint, Michigan, a postindustrial, urban community with high rates of preterm births, infant mortality, and child poverty, many women of child-bearing age carry the additional burden of previous lead exposure [11,12]. As a response to the lead-in-water public health crisis, a fruit and vegetable prescription program (FVPP) for pediatric patients was introduced and continues to provide one fresh fruit and vegetable prescription worth 15 US dollars to every patient (birth to 18 years) during each office visit. This program has demonstrated effectiveness in improving dietary patterns and household food security [13,14,15]. 

In 2022, the Flint Pediatric FVPP was expanded to reach pregnant women. This expansion was an intentional effort to address persistent barriers to access and affordability of high-nutrient foods necessary to support maternal and child health. Identical to the pediatric FVPP, the prenatal FVPP provides one prescription for fresh fruits and vegetables worth 15 US dollars at each prenatal visit (maximum of 14 prescriptions). The objective of the current report is to describe baseline sociodemographic characteristics, food security, and dietary intake in relation to US dietary guidelines of study participants prior to engagement in the prenatal FVPP. 

## 2. Materials and Methods

### 2.1. Setting

Approximately 45% of residents in Flint live in poverty, and local food stores offer lower-quality foods and fewer healthy food options than higher-income neighborhoods in the same county [4,5,6,16]. In addition to challenges related to healthy food access, the city recently endured a population-wide lead-in-water public health crisis [11]. 

### 2.2. Study Design

The study was a cross-sectional analysis of self-reported data from a convenience sample of pregnant women who presented for care at one of two obstetrics and gynecologic (OB/GYN) clinics in Flint, Michigan. Data were collected from pregnant women immediately prior to receiving their first prescription for fresh fruits and vegetables.

### 2.3. Prenatal Fruit and Vegetable Prescription Program

Using the electronic medical record system, paper prescriptions were ordered by physicians, printed, and distributed at the conclusion of office visits. Prescriptions may be redeemed at a local farmers’ market or a mobile market that delivers fresh produce boxes locally. Each patient received one fruit and vegetable prescription worth 15 US dollars at each prenatal visit, including the first postnatal visit (maximum of 14 total prescriptions).

### 2.4. Participants and Data Collection

After approval from Michigan State University Institutional Review Board (Study 00006239 title “Prenatal Nutrition Prescription Program”), all prenatal patients at two OB/GYN clinics received one prescription for fresh fruits and vegetables worth 15 US dollars. Patients were invited to participate in data collection if they were presenting for their first appointment, were between 18 and 43 years, and spoke English. Women with multiple or complicated pregnancies and women with severe pre-existing illnesses were excluded from analysis. Following written consent, participants completed survey questions using the secure digital platform, Research Electronic Data Capture (REDCap). This study was conducted in accordance with the Declaration of Helsinki.

### 2.5. Evaluation Tools

#### 2.5.1. Food Security

Participants completed the US Household Food Security Module: Six-Item Short Form (National Center for Health Statistics) to measure household food insecurity and hunger [17]. The sum of affirmative responses served as the household’s raw score (Table 1). Food security status was assigned based on a calculated raw score (0–1 = high/marginal food security; 2–4 = low food security; and 5–6 = very low food security).

#### 2.5.2. Dietary Intakes

Two 24 h dietary recalls were collected via the Automated Self-Administered 24-h (ASA-24) Dietary Assessment Tool developed by the National Cancer Institute [18]. The ASA-24 respondent website guided participants through the completion of each 24 h dietary recall. The first dietary recall was completed on the day of enrollment, and the second was completed within seven non-consecutive days. The ASA-24 researcher website was then accessed to obtain participant food group and nutrient data files. All dietary recall data were averaged over two days. 

#### 2.5.3. Food Access and Perceived Importance of Diet

To evaluate access to fruits and vegetables and related barriers, participants completed four questions from the Michigan Behavioral Risk Factor Surveillance Survey (MBRFSS). To assess perceived importance of nutrition and diet, participants responded to four additional questions designed to assess their perception of the importance of healthy eating. 

#### 2.5.4. Data Analyses

Descriptive analyses were conducted for maternal sociodemographic characteristics, food security, and dietary intake. Means with standard deviations or frequencies with percentages were calculated using IBM SPSS Statistics 27. Dietary intake data were reported in relation to US Dietary Guidelines for pregnancy. 

## 3. Results

### 3.1. Sociodemographic Characteristics

A total of 253 prenatal patients enrolled in the study. Most participants (mean age 26.5 ± 4.9 years) were African American (53.4%) and received publicly funded health insurance, i.e., Medicaid (66.4%). The majority (56.1%) reported having a high school degree or less with only 10.2% of the sample reporting graduation from college. A total of 105 participants (38.2%) were receiving assistance from the Special Supplemental Nutrition Program for Women, Infants, and Children (WIC), and 120 participants (43.6%) were receiving assistance from the Supplemental Nutrition Assistance Program (SNAP). 

### 3.2. Food Security

The majority of prenatal participants who completed the US Household Food Security Module (75.6%, *n* = 189) reported high/marginal food security. However, nearly 30% (*n* = 73) selected “often true” or “sometimes true” in response to the following statement: “The food that (I/we) bought just didn’t last, and (I/we) didn’t have the money to get more”. Approximately 10–15% of participants indicated “yes” when asked specific questions related to skipping meals, eating less because there was not enough money, or feeling hungry but not eating due to a lack of money for food (Table 1). 

### 3.3. Dietary Intake

Dietary intake was analyzed using the ASA-24 researcher website for food group and nutrient data files. A total of 144 participants (57%) completed two non-consecutive 24 h dietary recalls using ASA-24. An overwhelming majority of these participants failed to meet current dietary recommendations (Table 2). 

### 3.4. Food Access and Perceived Importance of Diet

Most respondents did not report challenges with food access, however, nearly 30% of participants acknowledged that distance to a full-service grocery store was “sometimes”, “usually”, or “always” a problem (Table 3). Nearly all participants acknowledged the importance of consuming a diet that includes fruits and vegetables (Table 3).

## 4. Discussion

This analysis describes dietary behaviors and food security among pregnant women in a low-income urban community immediately prior to the introduction of a prenatal fruit and vegetable prescription program. Central to our findings were notably poor dietary patterns among our entire sample of pregnant women. The majority of participants failed to meet dietary recommendations through food sources. This finding is consistent with the previous literature, demonstrating that prenatal diet quality is generally poor [19,20,21]. Moreover, study findings support research suggesting that there is a critical need to promote optimal nutrition among low-income African American women who are highly vulnerable to poor dietary habits during pregnancy [22,23]. 

Although most participants failed to meet dietary recommendations, a large majority acknowledged the importance of a healthy diet that includes fruits and vegetables during pregnancy. Moreover, most agreed that their doctors believed in the importance of a healthy diet. Previous research has suggested that pregnant women who are food insecure may default to cheap and convenient food choices even while recognizing the importance of eating healthy foods to support their pregnancy [24]. Related qualitative research has also suggested that although pregnant women have a general awareness of nutritional guidelines, too often they do not have the understanding or ability to put guidelines into practice [10]. These elusive challenges to healthy eating seemed to result in women feeling overwhelmed or confused with dietary recommendations throughout pregnancy [10,24]. Given the importance of optimal maternal nutrition for fetal and infant health [2,3], concentrated public health efforts should identify vulnerable patients who lack education and resources to support healthy eating during pregnancy and provide necessary resources. 

Unlike previous studies indicating that residents of low-income communities are often challenged with limited access to fresh, high-quality foods [7,25,26] participants in the current study reported few barriers to accessing fresh fruits and vegetables. In spite of poor fruit and vegetable consumption among study participants, most were satisfied with both the availability and quality of fruits and vegetables while shopping. Additionally, most study participants reported high or marginal food security. It is important to note, however, that nearly 30% of participants indicated that distance from their homes to a full-service grocery store was difficult. Previous studies pointed to transportation as a persistent barrier to healthy food access in low-income communities [27,28]. 

Limitations of the current study include results specific to one low-income urban community, potentially limiting generalizability. Selection bias cannot be ruled out, as responses from pregnant women who chose to participate in the study may have differed from those who chose not to participate, although characteristics of those who participated in the current study closely matched characteristics of the source population at the partnering clinics. Dietary intake data were collected via 24 h dietary recall, which is the gold standard for dietary intake data; however, the two 24 h recalls we were able to collect may not be representative of usual intake for each participant. The design of the current study did not allow for investigation of the impacts of the prenatal FVPP. 

## 5. Conclusions

Although prior evidence demonstrates the critical importance of proper nutritional status before and during pregnancy to support optimal fetal growth and brain development [3], the current study suggests that pregnant women in a US city are not meeting dietary recommendations. These findings raise concerns regarding diet and nutrition among pregnant women in Flint, Michigan, and indicate a need for diet assessment to occur more broadly during pregnancy to create awareness of the potential for poor nutritional status during this critical life stage. There is an urgent need to develop and implement programs that address persistent barriers to healthy eating, which exacerbate the negative impacts of public health disasters [12,29,30,31]. Future research will investigate whether the prenatal FVPP, which serves as a tangible resource for physicians to address nutrition security, is effective in addressing poor dietary patterns among prenatal patients.

## Figures and Tables

**Table 1:** Household food security.

Food Security Status (Score) ^a^		Frequency (*n* = 250)
High/Marginal food security (0–1)		75.6% (*n* = 189)
Low food security (2–4)		13.2% (*n* = 33)
Very low food security (5–6)		11.2% (*n* = 28)
**Screener Question**	**Response ^b^**	**Frequency (*n* = 250)**
1. The food that (I/we) bought just didn’t last, and (I/we) didn’t have the money to get more.	Often true/Sometimes true	29.2% (*n* = 73)
2. (We) couldn’t afford to eat balanced meals.	Often true/Sometimes true	29.6% (*n* = 74)
3. In the last 12 months, did (you/you or other adults in your household) ever cut the size of your meals or skip meals because there wasn’t enough money for food?	Yes	13.2% (*n* = 33)
3a. How often did this happen?	Almost every month/Some months but not every month	11.2% (*n* = 28)
4. In the last 12 months, did you ever eat less than you felt you should because there wasn’t enough money for food?	Yes	15.2% (*n* = 38)
5. In the last 12 months, were you ever hungry but didn’t eat because there wasn’t enough money for food?	Yes	10.8% (*n* = 27)

^a^ Assessed with US Household Food Security Survey Module: Six-Item Short Form [17] (possible scores 0–6; lower = greater food security). ^b^ Responses of “Often true” or “Sometimes true” on questions 1 and 2 and “Yes” on 3, 4, and 5 are coded as affirmative (yes = 1). Responses of “Almost every month” and “Some months but not every month” on question 3a are coded as affirmative (yes = 1). The sum of affirmative responses to the six questions is the household’s raw score on the scale.

**Table 2:** Participant food group and nutrient intakes in comparison to US Dietary Recommendations (*n* = 144 participants; dietary intake variables all represent average of two dietary recalls).

Food Group Intake	US Recommendations (Women 19–30 Years Old)	Percent Meeting Current Dietary Recommendations	Mean ± SD (*n* = 144)
Fruits (cup eq.)	1 ½ to 2 cups	30.6%	1.09 ± 1.07
Vegetables (cup eq.)	2 ½ to 3 cups *	8.3% *	1.24 ± 1.17
Protein (g)	71 g	45.8%	71.18 ± 29.82
Dairy (cup eq.)	3 cups	6.9%	1.36 ± 0.88
Whole grains (oz eq.)	3 to 4 oz **	0.7%	0.53 ± 0.68
**Nutrient Intake**	**US Dietary Recommendations (Pregnant Person 19–50 y)**	**Percent Meeting Current Dietary Recommendations**	**Mean ± SD** **(*n* = 144)**
Calcium (mg)	1000 milligrams ***	31.2%	871.10 ± 383.01
Iron (mg)	27 milligrams	1.4%	13.03 ± 6.37
Vitamin A (mcg)	770 micrograms ****	17.4%	548.45 ± 356.58
Vitamin C (mg)	85 milligrams *****	37.5%	85.27 ± 74.08
Vitamin D (mcg)	600 international units	0%	128.40 ± 93.6
Folic acid (mcg)	600 micrograms	1.4%	178.67 ± 129.82

* For women 31–59 years old, daily vegetable recommendation is 2 to 3 cups. Note: two participants 31 years old or older reported an average of 2 cups of vegetables; ** For women 31–58 years old, daily whole grains recommendation is 3 to 3.5 oz.; *** Acog.org/womens-health/faqs/nutrition-during-pregnancy (24 January 2024) 1300 milligrams of calcium for 14–18 years old; **** 750 micrograms of vitamin A for 14–18 years old; and ***** 80 milligrams of vitamin C for 14–18 years old.

**Table 3:** Participant-reported food access and perceived importance of diet.

Question	Response	*n*	Frequency
Food Access ^a^			
How often does the distance from your home to a full-service grocery store make it difficult for you to buy the variety and quality of fruits and vegetables you would like?	Always, Usually, Sometimes	238	29.0%
How often is a variety of good quality dark green vegetables, such as broccoli, romaine, chard, collard greens, or spinach, available at this location?	Always, Usually, Sometimes	238	94.1%
How often is a variety of good quality orange-colored vegetables, such as sweet potatoes, pumpkin, winter squash, or carrots, available at this location?	Always, Usually, Sometimes	234	89.7%
How often is a variety of fresh, frozen, or canned fruits available at this location?	Always, Usually, Sometimes	238	93.7%
**Perceived Importance of Diet ^b^**			
It is important to eat a healthy diet that includes fruits and vegetables.	Agree or Strongly Agree	247	87.4%
A diet that includes fruits and vegetables is especially important during pregnancy.	Agree or Strongly Agree	245	90.6%
A diet that includes fruits and vegetables is especially important for children.	Agree or Strongly Agree	245	91.8%
My doctors think it is important for me to eat a healthy diet that includes fruits and vegetables.	Agree or Strongly Agree	243	90.1%

^a^ All response options: “Always”, “Usually”, “Sometimes”, “Rarely”, and “Never”. Responses of “Always”, “Usually”, or “Sometimes” indicate greater self-reported food access. ^b^ All response options: “Strongly Agree”, “Agree”, “Neutral”, “Disagree”, and “Strongly Disagree”. Responses of “Strongly Agree” or “Agree” indicate greater perceived importance of diet.

## Data Availability

Data are available upon request from the corresponding author.
